# Discriminatory performance of adiponectin and leptin in the identification of impaired glucose tolerance: The Guangzhou Biobank Cohort Study - Cardiovascular Disease Subcohort

**DOI:** 10.1371/journal.pone.0206964

**Published:** 2018-11-06

**Authors:** Konstantinos A. Toulis, Chao Q. Jiang, Karla Hemming, Krishnarajah Nirantharakumar, Kar K. Cheng, Tai H. Lam, G. Neil Thomas

**Affiliations:** 1 Institute of Applied Health Research, University of Birmingham, Birmingham, United Kingdom; 2 Department of Endocrinology, Thessaloniki, Greece; 3 Guangzhou Occupational Disease Prevention and Treatment Centre, Guangzhou, People's Republic of China; 4 Department of Community Medicine, University of Hong Kong, Pokfulam, Hong Kong SAR, People's Republic of China; 5 Institute of Public Health, Social and Preventive Medicine, Mannheim Medical Faculty, Heidelberg University, Mannheim, Germany; Institute of Health Economics and Health Care, GERMANY

## Abstract

**Background:**

To evaluate the additional discriminatory performance of adiponectin, leptin, and their ratio in the identification of impaired glucose tolerance (IGT) in men and women without diabetes on top of conventional risk factors.

**Methods & results:**

A total of 698 subjects underwent an oral glucose tolerance test (oGTT) and adipocytokine measurements. A comprehensive stepwise selection procedure was performed, followed by c-statistics and integrated discrimination improvement (IDI) analysis. In males, adiponectin levels were significantly lower in the IGT group compared to the non-IGT group (Whitney U test, p < 10–4), whereas leptin levels were significantly higher (p = 0.009) in IGT group. In females, adiponectin and leptin levels were not significantly different between groups (Mann-Whitney U test, p = 0.073 and p = 0.08, respectively). Adjusting for the most informative, sex-specific, clinical and biochemical factors, adiponectin, leptin and their ratio were not found to be significant predictors of the response to the glucose load, when modelled as continuous terms or tertiles. In males, the area-under-the-curve (AUC) for adiponectin was estimated at 0.620 (95% CI: 0.558–0.682) and the addition of adiponectin into the basic model provided a ΔAUC benefit of 0.004, showing no additional discriminatory benefit on top of conventional risk factors (IDI p-value: 0.27), nor did the addition of leptin or their ratio. The results were similar in females.

**Conclusions:**

In Chinese individuals without diabetes, no significant evidence for the potential discriminatory value of adiponectin, leptin or their ratio in the identification of IGT on top of conventional risk factors was observed.

## Introduction

Insulin resistance (IR) is closely associated with abdominal adiposity, a surrogate measure of visceral adiposity. Adipocytes are considered to be hormonally active by releasing two proteins, adiponectin and leptin. Adiponectin is thought to exert insulin-sensitizing, anti-atherogenic and anti-inflammatory actions [1; 2] via peroxisome-proliferator receptor γ action [3]. Leptin, a protein circulating in proportion with body fat-cell stores, reflects nutritional status and subcutaneous fat mass, information essential for the regulation of appetite and energy expenditure by neural centres [4–6].

Interestingly, adiponectin has been found to be inversely and independently associated with IR [7; 8] and impaired glucose tolerance (IGT) using a range of insulin sensitivity indices [9]. A meta-analysis of prospective cohort studies confirmed that higher adiponectin concentrations were associated with a lower risk of type 2 diabetes mellitus (T2DM) [10]. Conversely, leptin has been reported to have a positive correlation with IR [11]. The leptin: adiponectin ratio has previously been suggested to be a better indicator of IR than the individual adipokines [12].

Despite evidence describing the associations between adiponectin, leptin and their ratio with risk of T2DM, evidence regarding their clinical utility is still unclear [10; 13; 14]. Most importantly, the literature on the performance in correctly identifying subjects with glucose tolerance status is still scarce and contradictory [15; 16]. This may be of particular concern in terms of preventing cardiovascular disease (CVD) risk, given the role of postprandial glycaemia on both the onset of CVD [17] and secondary CVD prevention [18]. Moreover, ethnic variances in adipokine have been reported and Asians may have the least favorable profile [19; 20]. Finally, it has been demonstrated in a cluster randomized trial that lifestyle intervention programme for Chinese people with impaired glucose tolerance can reduce incidence of cardiovascular and all-cause mortality and diabetes over a period of 23 years [21]. Therefore, timely identification of the prediabetic state may be an issue of major public health importance.

Thus, the present study was aimed to evaluate (1) the diagnostic performance of adiponectin, leptin and their ratio in the correct classification of glucose intolerance status in men and women using oral glucose tolerance test (oGTT) as the reference standard, and (2) whether adipokine levels would enhance the discriminatory value of established markers in predicting impaired glycaemic status. A positive result would be a proof of their still uncertain clinical utility and an alternative for the poorly reproducible oGTT.

## Methods

### Subjects and measurements

The Guangzhou Biobank Cohort Study is an ongoing collaboration between the Guangzhou Number 12 Hospital, Guangzhou, China, the University of Hong Kong, Hong Kong, and the University of Birmingham, Birmingham, U.K. The Guangzhou Biobank Cohort Study–Cardiovascular Disease Subcohort (GBCS-CVD) consists of 1,300 participants who were intensively phenotyped for a range of surrogate markers of vascular disease as well as coagulatory and inflammatory markers [22]. A random subset of the participants (n = 698) underwent both adipokine measurement and oGTT, which was interpreted according to relevant guidelines [23]. Patients with known diabetes mellitus were excluded from the analysis. Those with a normal response to glucose load, yet with impaired fasting glycaemia (IFG, fasting glucose > 5.6 and < 7.0 mmol/L) were included in the non-IGT group for the purpose of analysis and this inclusion was then explored in sensitivity analyses. Those patients with a response to oGTT in the non-diabetic range and with a glycated haemoglobin (HbA1c) measured in the diabetic range (HbA1c > 6.5%) were initially included in the study group, yet subsequently excluded in the sensitivity analyses. Detailed descriptions of the measurement methodology can be found elsewhere [22; 24]. The Guangzhou Biobank Cohort Study was approved by Guangzhou Medical Ethics Committee of the Chinese Medical Association, Guangzhou, China and written informed consent was obtained from all participants.

### Statistical analysis

Subject characteristics were summarised by glycaemic status, using means and standard deviations, medians and inter-quartile ranges or numbers and percentages as appropriate. Normality was ascertained by visualisation and confirmed by Shapiro-Wilk test. Unadjusted odds ratios (IGT vs. non IGT) were then computed along with 95% confidence intervals. The non-parametric Mann-Whitney U test was also used to examine differences between the groups. All analyses were stratified by sex.

To select important risk factors (not including adiponectin, leptin and their ratio), including anthropometric measurements, age, parental history, lipids, inflammation markers for adjustment we used a comprehensive stepwise selection procedure [25]. This procedure involved selecting for possible inclusion from potential variables the subset of covariates for which the P-value for the unadjusted odds ratio was less than 0.25. These covariates were then included in a forward stepwise selection procedure, with P-value for addition set at ≤0.05 and the P-value for removal set at ≥0.1. This was then complemented by carrying out a sensitivity check, by implementing a backwards stepwise selection procedure with P-value for addition ≤0.05 and P-value for removal ≥0.1. Any covariates which were selected for inclusion in the backwards method but not selected in the forwards method, were then added to the model selected using the forwards stepwise procedure and retained if their P-value was less than 0.1. Finally, this procedure was augmented by adding any covariates not selected so far into the model, one at a time, and retaining if their P-value was less than 0.1. All levels of categorical variables were included if one level met the inclusion criteria. Finally, to mitigate against over fitting, bootstrapping was then performed to reduce optimism and shrinkage estimates reported.

The objective was to determine the prognostic value of adiponectin and leptin, in addition to the prognostic value obtained from conventional factors (i.e. by the model selected directly above). To this end, adiponectin and leptin, individually and jointly, and the ratio of leptin to adiponectin, were added to the model selected above, to determine their predictive effects after adjustment for conventional risk factors. These factors were included firstly as continuous variables and then as categorical variables (tertiles) to allow for departures of non-linear effects.

Predictive performance of models was compared by computing c-statistics [area under the curve (AUC)] and calculating the integrated discrimination improvement (IDI). The latter can be interpreted as a difference in discrimination slopes between models (difference of mean predicted probabilities of events and non-events) and a significant result (P-value < 0.05) denotes substantial improvement [26]. Model calibration was assessed by computing the Hosmer and Lemeshow goodness of fit test (a P-value > 0.05 was considered indicative of an adequate fit). All continuous variables were checked for normality and those for which there was evidence of a departure from normality were transformed using an appropriate transformation. All continuous variables were then standardized onto the z-scale by subtracting the mean and dividing by the standard deviation. This means that all OR for continuous variables can be interpreted as increase in odds per standard deviation. No data were missing and no imputation analyses were performed. All analyses were carried out in Stata 11.2 SE.

## Results

Out of 698 subjects (354 males, 344 females), one hundred ninety-six (106 males and 90 females) subjects were found to have IGT. Gender-specific sample characteristics, along with results from univariate logistic regression analyses, are presented in Table 1. Sex-specific AUCs for the prediction of IGT status using adiponectin, leptin or their ratio were calculated and presented along with AUCs for other predictors and that of basic model, consisted of the conventional predictors selected through the variable selection strategy (Table 2). Of note, AUC for adiponectin was generally not inferior compared to the AUCs of the most informative clinical and biochemical markers in males without diabetes.

**Table 1:** Sex-specific characteristics and results from univariate and multivariate logistic regression analyses.

	Non-IGT group	IGT group	Crude Odds Ratio (95% CI)	Adjusted odds ratio (95% CI)	Non-IGT group		IGT group	Crude Odds Ratio (95% CI)	Adjusted odds ratio (95% CI)
	Males (n = 354)				Females (n = 344)				
N (%)	248 (70.05)	106 (29.94)			254 (73.8)	90 (26.2)			
*Adipocytokines*									
Adiponectin(μg/ml)	5.76 [6.13]	4.31 [3.20]	0.76 (0.61–0.96)		7.48 [8.78]	6.58 [6.07]		0.84 (0.68–1.05)	
Leptin (ng/ml)	3.59 [4.74]	4.93 [4.87]	1.34 (1.06–1.70)		11.2 [11.81]	13.3 [9.79]		1.29 (0.93–1.81)	
Leptin:Adiponectin Ratio	0.55 [1.06]	1.09 [1.34]	1.45 (1.14–1.50)		1.54 [2.70]	1.89 [2.20]		1.21 (1.01–1.45)	
*Anthropometric data*									
Body mass index (kg/m2)	22.6 [3.66]	24.3 [3.52]	1.72 (1.34–2.19)	1.47 (1.05–2.04)	23.0 [3.64]	23.8 [3.77]		1.31 (1.02–1.67)	
Waist(cm)	78.5 [11.0]	84.0 [11.0]	1.70 (1.33–2.17)		74.0 [10.0]	78.0 [10.0]		1.50 (1.17–1.92)	
Waist:hip ratio	0.89 [0.08]	0.91 [0.06]	1.56 (1.22–1.99)		0.82 [0.08]	0.85 [0.10]		1.65(1.26–2.17)	1.49 (1.07–2.08)
*Glucose and insulin*									
Haemoglobin A1c (%)	5.90 [0.50]	6.0 [0.60]	1.35 (1.04–1.76)		5.81 [0.4]	6.0 [0.50]		1.58 (1.19–2.10)	
Glucose at 0h(mmol/L)	5.11 [0.60]	5.54 [0.70]	1.67 (1.29–2.17)	3.36 (1.62–6.95)	5.02 [0.52]	5.40 [0.58]		1.82 (1.40–2.37)	3.65 (2.33–5.74)
Glucose at 2h(mmol/L)	6.23 [1.64]	9.12 [1.31]			6.34 [1.3]	8.59 [1.14]			
Insulin at 0h(mU/L)	4.54 [4.05]	6.18 [5.81]	1.74 (1.16–2.60)		5.84 [3.89]	7.02 [5.73]		1.17 (0.87–1.58)	
Insulin at 2h(mU/L)	16.2 [17.6]	32.7 [36.2]			19.6 [17.8]	36.6 [43.2]			
HOMA-IR	1.05 [0.99]	1.41 [1.47]	1.91 (1.24–2.97)		1.34 [1.04]	1.79 [1.4]		1.22 (0.89–1.67)	
*Lipids*									
Triglycerides (mmol/L)	1.35 [0.98]	1.70 [1.14]	1.41 (1.12–1.77)		1.2 [0.75]	1.54 [1.02]		1.37 (1.08–1.75)	
LDL(mmol/L)	3.11 [0.84]	3.19 [0.57]	1.02 (0.81–1.28)		3.43 [0.96]	3.54 [0.56]		1.11 (0.87–1.41)	
HDL(mmol/L)	1.39 [0.43]	1.27 [0.38]	0.66 (0.52–0.85)	0.70 (0.50–0.97)	1.71 [0.52]	1.64 [0.45]		0.84 (0.65–1.08)	
Total cholesterol (mmol/L)	5.44 [1.32]	5.41 [1.26]	0.98 (0.78–1.23)		5.91 [1.35]	6.22 [1.42]		1.76 (0.51–6.09)	
TCHDL ratio	3.80 [1.26]	4.30 [1.48]	1.44 (1.14–1.82)		3.48 [1.32]	3.73 [1.34]		1.19 (0.93–1.52)	0.60 (0.37–0.95)
Apolipoprotein B (mmol/L)	0.94 [0.36]	0.99 [0.33]	1.10 (0.88–1.38)		1.03 [0.39]	1.07 [0.43]		1.33 (1.03–1.72)	2.13 (1.31–3.47)
*Inflammation markers*									
hsCRP(mg/L)	1.26 [2.3]	1.49 [2.11]	1.15 (0.95–1.40)		0.97 [2.25]	1.79 [2.54]		1.43 (1.10–1.82)	
PAI-1(ng/ml)	179 [179]	194 [162]	1.14 (0.87–1.49)		144 [137]	187 [172]		1.22 (0.88–1.70)	
Interleukin-6 (ng/ml)	19.7 [10.9]	20.0 [12.9]	0.85 (0.63–1.17)		18.2 [12.65]	20 [9.85]		1.03 (0.81–1.31)	
*Others*									
Age(years)	61.0 [9.0]	61.5 [9.0]	1.14 (0.91–1.42)		54.0 [4.0]	55.0 [6.0]		1.33 (1.06–1.68)	
Aspartate aminotansferase	24.1 [8.03]	24.0 [8.70]	0.86 (0.81–1.28)		23.4 [6.75]	23.4 [7.65]		1. 19 (0.50–2.85)	
Alanine aminotansferase	21.0 [13.3]	24.8 [15.5]	1.35 (1.07–1.70)		18.4 [10.8]	21.1 [12.3]		1.21(0.81–1.31)	
SBP(mmHg)	123 [26.7]	132 [23.8]	1.54 (1.21–1.95)	1.47 (1.02–1.91)	116 [26.7]	126 [27.3]		1.80 (1.38–2.34)	1.76 (1.28–2.43)
DBP(mmHg)	73.0 [13.6]	76.0 [13.3]	1.45 (1.14–1.83)		69.5 [16]	73.5 [13.5]		15.1 (1.16–1.93)	
Parental history of diabetes (%)	7 (3.6)	7 (6.6)	1.64 (0.61–4.42)		25 (11.7)	9 (10.1)		0.84 (0.38–1.89)	
Physical activity (%)	107 (55.4)	55 (53.9)	0.94 (0.60–1.62)		140 (67.0)	56 (64)		1.01 (0.69–1.48)	

Variables are presented as median [interquartile range] or as N (%). All odds ratios for continuous variables should be interpreted as increase in odds per standard deviation. Subjects were designated with physical activity if they were classified into the high category was on the basis of International Physical Activity Questionnaires (IPAQ).

CI: Confidence interval, DBP: Diastolic blood pressure, HDL: High-density lipoprotein, HOMA-IR: Homeostasis model assessment–insulin resistance, hsCRP: high-sensitive C- reactive protein, IGT: Impaired glucose tolerance, LDL: Low-density lipoprotein, PAI-1: Plasminogen activator inhibitor -1, SBP: Systolic blood pressure, TCHDL ratio: Total cholesterol-to-high-density lipoprotein ratio

**Table 2:** Areas under the curve (AUC) and 95% confidence intervals (CIs) from receiver-operator characteristics (ROC) analysis for the prediction of impaired glucose tolerance (conventional predictors).

		AUC	95% CI		AUC	95% CI
	**Males**			**Females**		
Adiponectin		0.620	0.558–0.682		0.436	0.369–0.503
Leptin		0.587	0.524–0.650		0.562	0.496–0.627
Leptin:Adiponectin		0.633	0.571–0.695		0.580	0.517–0.644
BMI		0.653	0.592–0.714		0.589	0.521–0.657
Waist		0.646	0.585–0.707		0.618	0.552–0.683
Glucose at 0h		0.664	0.602–0.725		0.663	0.597–0.728
Insulin at 0h		0.641	0.573–0.709		0.565	0.488–0.642
HbA1c		0.585	0.519–0.651		0.622	0.553–0.691
Triglycerides		0.602	0.544–0.669		0.604	0.534–0.673
HDL		0.628	0.565–0.691		0.460	0.392–0.528
ALT		0.588	0.523–0.653		0.564	0.498–0.631
SBP		0.636	0.575–0.697		0.657	0.591–0.772
Parental history of diabetes mellitus		0.512	0.446–0.579		0.493	0.457–0.531

In males, adiponectin levels were significantly lower in the IGT (cases) group as compared to the non-IGT (control) group (Whitney U test, *p* < 10^−4^), whereas leptin levels and leptin:adiponectin ratio were significantly higher (*p* = 0.009 and *p* < 10^−4^ respectively) in IGT group (Table 1). Table 3 shows that the AUC for the prediction of IGT with adiponectin was estimated at 0.62 (95% CI: 0.56–0.68) in males. Table 4 shows that when modelled as a continuous term adjusting for established conventional factors as selected from the stringent variable selection strategy [fasting glucose, body mass index (BMI), systolic blood pressure, high-density lipoprotein (HDL)-cholesterol], adiponectin was not found to be a significant predictor of IGT status [0.86 (0.67–1.10)]. To test for non-linear effects, adiponectin tertiles were also examined and again found to be non-significant. Leptin, leptin:adiponectin and both adiponectin and leptin also did not show any statistical significance (Table 4). The addition of adiponectin essentially showed no discriminatory benefit when compared to the model consisted of traditional markers in terms of AUC (ΔAUC = 0.004, IDI estimate: 0.003, *p*-value: 0.27), neither did the addition of leptin (IDI *p*-value: 0.18) or their ratio (IDI *p*-value: 0.09) (Table 3). Sensitivity analyses (1) with the exclusion of patients with IFG and (2) with the exclusion of those patients with HbA1c ≥ 6.5% but with a response to oGTT in the non-diabetic range did not alter the findings (data not shown).

**Table 3:** Areas under the curve (AUC) and 95% confidence intervals (CIs) from receiver-operator characteristics (ROC) analysis for the prediction of impaired glucose tolerance.

	AUC	95% CI	IDI estimates	IDI p-value
**Males**				
Basic model	0.728	0.671–0.786	n/a	n/a
Basic model + Adiponectin	0.731	0.674–0.789	0.00333	0.27
Basic model + Leptin	0.731	0.673–0.789	0.00301	0.18
Basic model + Leptin:Adiponectin	0.729	0.671–0.786	0.00017	0.09
Basic model + Adiponectin + Leptin	0.734	0.676–0.791	0.00634	0.90
**Females**				
Basic model	0.756	0.697–0.816	n/a	n/a
Basic model + Adiponectin	0.760	0.701–0.819	0.00374	0.28
Basic model + Leptin	0.756	0.697–0.816	0.00123	0.53
Basic model + Leptin:Adiponectin	0.758	0.699–0.818	0.00095	0.59
Basic model + Adiponectin + Leptin	0.761	0.702–0.820	0.00415	0.19

The basic Model was constructed using the variables: Glucose, body mass index (BMI), systolic blood pressure (SBP), high-density lipoprotein (HDL) for males and waist:hip ratio, Glucose, SBP, apolipoprotein B and total cholesterol:hdl ratio for females. Each Area-under-the-Curve (AUC) was compared to that of the basic models.

ALT: Alanine aminotansferase, CI: Confidence interval, Exp: Exponential, HbA1c: Haemoglobin A1c, IDI: Integrated Discrimination Improvement

**Table 4:** Sex-specific odds ratios and 95% confidence intervals (CIs) for impaired glucose tolerance prediction according to adipocytokine levels adjusting for conventional factors.

	Continuous term	T1	T2	T3
**Adiponectin**				
Males	0.86 (0.67–1.10)	1.76 (0.90–3.41)	1.64 (0.86–3.16)	reference
Females	0.85 (0.63–1.14)	1.18 (0.59–2.38)	1.56 (0. 79–3.09)	reference
**Leptin**				
Males	0.89 (0.65–1.21)	reference	1.03 (0.54–1.96)	0.76 (0.37–1.56)
Females	0.90 (0.66–1.22)	reference	1.39 (0.71–2.76)	0.74 (0.36–1.52)
**Leptin:Adiponectin ratio**				
Males	1.08 (0.80–1.45)	reference	1.49 (0.76–2.91)	1.65 (0.79–3.47)
Females	1.10 (0.82–1.46)	reference	2.11 (1.06–4.19)	1.26 (0.62–2.57)

The basic model (conventional factors) was constructed using the variables fasting glucose (Glu), body mass index (BMI), systolic blood pressure (sbp), high density lipoprotein (HDL) for males and waist:hip ratio, Glu, sbp, apolipoprotein B and total cholesterol:hdl ratio for females

Adip:Adiponectin, Lept: Leptin, T: Tertile

In females, there was no evidence that adiponectin and leptin levels were different between groups (Mann-Whitney U test, *p* = 0.073 and *p* = 0.08, respectively), but leptin:adiponectin ratios differed significantly (Mann-Whitney U test, *p* = 0.023). Adjusting for waist:hip ratio, fasting glucose, total cholesterol: HDL cholesterol ratio and apolipoprotein, none of the adipocytokines were significant predictors of oGTT-based classification (Table 4) or conferred any discriminatory benefit in addition to that of the basic model (Table 3). Finally, analysis using tertiles of adiponectin, leptin or their ratio adjusting for the selected conventional factors showed similar negative findings (Table 4). Sensitivity analyses did not alter the findings (data not shown).

## Discussion

In this cross-sectional analysis, although the AUC for adiponectin in regards to IGT prediction was generally not inferior compared to the AUCs of the most informative clinical and biochemical markers in males non-diabetics, it was rather modest (0.62) and the inclusion of adiponectin did not have discernible benefit over that of the conventional risk factors. Furthermore, there was no evidence to suggest that adiponectin, leptin or their ratio were significant predictors of the response to the oral glucose load. They showed no substantial discriminatory value in identifying IGT in subjects without diabetes when conventional predictors were taken into account. This finding was robust in all analyses, either when the adipocytokines were analysed as continuous variables or in tertiles. Of note, this study was not design to assess the predictive ability of adipocytokines in the subsequent development of T2DM.

The pathophysiology underlying the previously reported association in the literature is not fully elucidated [27]. It appears that adiponectin improves insulin sensitivity by increasing fatty acid oxidation in muscle, and affects glucose homeostasis by suppressing hepatic glucose production [1]. In addition to these peripheral actions, adiponectin might have direct effects on beta cells and thus could be considered as a positive, dual regulator of both insulin sensitivity and secretion [5]. Moreover, the pattern of sex differences, the so-called “sexual dimorphism” in adiponectin, which might be explained on the basis of different fat amount and the influences of sex hormones [28], was also observed in the present study and generally in the literature [29; 30]. Failure of leptin to demonstrate discriminative power in IGT prediction may be explained, in part, by the observation that the effects of leptin on glucose metabolism are much less pronounced in human skeletal muscle compared with results from animal studies and by the hypothesis that in humans, leptin may hold a rather regulatory than instant-acting role in the “adipoinsular axis” [6]. Impaired fasting glycaemia is a rather multifaceted condition, a global indicator of suboptimal glucose handling, potentially reflecting several different underlying pathophysiologies, ranging from decreased beta-cell mass to insulin resistance or genetic defects. Thus, another potential explanation for the negative findings is the heterogeneous nature of the condition.

People with IGT represent an asymptomatic subpopulation with increased CVD risk [31] and there is evidence to suggest that increased risk for CVD observed in IGT is due, at least in part, to lower-than-normal levels of adiponectin [27; 32]. Ko *et al* investigated the discriminatory performance of adiponectin jointly with waist circumference in middle-aged males [15]; yet, that analysis did not include females or leptin. Also, ROC analysis was reported only for diabetes prediction [AUC = 0.642) and not for IGT and most importantly, potentially important confounders such as fasting glucose, insulin, HbA1c or inflammation markers were not taken into consideration. Wu *et al* also provided an evaluation of the discriminatory ability of several biomarkers, including adiponectin (AUC = 0.64 for diabetes prediction), using a weighted biomarker risk score [14]; however, the analysis did not account for fasting glucose, insulin and HbA1c, and an oGTT was not performed. Kolberg *et al* provided an elegant assessment of the discriminatory ability of six selected biomarkers, including adiponectin, for the prediction of diabetes, but they did not include IGT as an outcome [13].

The present study had several limitations. First, high-molecular weight (HMW) adiponectin, the “active” adiponectin, was not investigated, preventing the confirmation of the reported lack of the oGTT-induced transient decrease in HMW adiponectin in subjects with IGT [33]. Secondly, it should be noted that 84 subjects with IFG (46 males and 38 females) were included into the normal glucose tolerance group. This decision was based on the distinct aetiologies between IGT and IFG [34], the essentially differential roles of incretin hormones and insulin action [35] and the disparate pattern of HMW adiponectin response to an oral glucose load [33]. However, exclusion of these subjects did not change the results.

In summary, the role of adipocytokines in the detection of IGT was explored in a well characterized group of Chinese individuals without diabetes. Through a detailed analysis adjusting for multiple potential confounders, no significant evidence for their potential discriminatory value in addition to conventional risk factors in the identification of IGT was observed.
